# Shock in the emergency department; a 12 year population based cohort study

**DOI:** 10.1186/s13049-016-0280-x

**Published:** 2016-06-30

**Authors:** Jon Gitz Holler, Daniel Pilsgaard Henriksen, Søren Mikkelsen, Lars Melholt Rasmussen, Court Pedersen, Annmarie Touborg Lassen

**Affiliations:** Department of Emergency Medicine, Odense University Hospital, Sdr Boulevard 29, Entrance 130, 1. Floor 5000, Odense C, Denmark; Department of Respiratory Medicine, Odense University Hospital, Odense C, Denmark; Department of Anesthesiology and Intensive Care Medicine, Odense University Hospital, Odense C, Denmark; Centre for Individualized Medicine in Arterial Diseases (CIMA) Department of Clinical Biochemistry and Pharmacology, Odense University Hospital, Odense C, Denmark; Department of Infectious Diseases, Odense University Hospital, Odense C, Denmark

**Keywords:** Shock, Epidemiology, Incidence, Mortality

## Abstract

**Background:**

The knowledge of the frequency and associated mortality of shock in the emergency department (ED) is limited. The aim of this study was to describe the incidence, all-cause mortality and factors associated with death among patients suffering shock in the ED.

**Methods:**

Population-based cohort study at an University Hospital ED in Denmark from January 1, 2000, to December 31, 2011. All patients aged ≥18 years living in the hospital catchment area with a first time ED presentation with shock (*n* = 1646) defined as hypotension (systolic blood pressure (SBP) ≤100 mmHg)) and ≥1 organ failures. Outcomes were annual incidence per 100,000 person-years at risk (pyar), all-cause mortality at 0–7, and 8–90 days and risk factors associated with death.

**Results:**

We identified 1646 of 438,191 (0.4 %) ED patients with shock at arrival. Incidence of shock increased from 53.8 to 80.6 cases per 100,000 pyar. The 7-day, and 90-day mortality was 23.1 % (95 % CI: 21.1–25.1) and 40.7 % (95 % CI: 38.3–43.1), respectively. Independent predictors of 7-day mortality were: age (adjusted HR 1.03 (95 % CI: 1.03–1.04), and number of organ failures (≥3 organ failures; adjusted HR 3.13 95 % CI: 2.28–4.30). Age, comorbidity level and number of organ failure were associated with 90-day mortality.

**Conclusion:**

Shock is a frequent and critical finding in the ED, carrying a 7- and, 90- day mortality of 23.1 and 40.7 %, respectively. Age and number of organ failures are independent prognostic factors for death within 7 days, whereas age, comorbidity and organ failures are of significance within 8–90 days.

## Background

Shock is a life-threatening condition of circulatory failure that requires prompt recognition, diagnosis, and resuscitation [1]. It is a substantial cause of morbidity and mortality and is associated with high healthcare costs [1, 2].

Although the majority of critically ill patients are identified and initially resuscitated in the Emergency Department (ED) setting, the knowledge of outcomes and the epidemiological characteristics of shock has traditionally been limited to the post ED-period [3]. As these studies are based on different populations sampled several hours after the initial ED identification and resuscitation, the estimates are of limited value for understanding the early characteristics at presentation in the ED.

While trends in frequency and mortality of undifferentiated ED shock are largely unexplored, the few studies available report in-hospital mortality of up to 24 % in US ED settings [4, 5]. Despite the substantial mortality reported, there is limited information on the epidemiological characteristics of shock from a population-based perspective. Clarifying the epidemiology of shock at presentation in the ED, in a population-based context, are critical steps to uncover the full burden of shock in the pre-intensive care unit (ICU) period.

The aim of the present study was to examine the epidemiological characteristics of shock in an ED setting in Denmark. Our primary objective was to examine the 7- and 90- day all cause mortality of patients arriving to the ED in Odense University Hospital during the period 2000–2011. Secondary, factors associated with death were explored, as well as trends in annual incidence and mortality.

## Methods

### Study design and setting

We conducted a population-based cohort study in patients treated at the ED at Odense University Hospital, Denmark, between 1st January 2000 - 31th December 2011. This ED serves a mixed rural-urban population of 225,000 person (age ≥18) and provides 24-hour acute medical care with 37,000 annual adult visits. Odense University Hospital is a 1000-bed university teaching hospital that serves as the only primary hospital for the local community as well as a Level 1 trauma center with all specialties represented (see Table 1). At Odense University Hospital patients are usually assessed in the ED and hereafter allocated and admitted to one of the specialties presented in Table 1 or referred to primary care after primary ED evaluation. In the prehospital setting, the basic response to a request of prehospital assistance is an ambulance staffed by two emergency medical technicians (EMTs) [6]. The competences are restricted to initial treatment of patients with myocardial infarction (nitroglycerine, thrombolytic agents, opioids), fluid administration and defibrillation, as well as inhalational therapy, rectal administration of benzodiazepines, intramuscular administration of naloxone and adrenaline [6]. From 2006 and onwards a physician-staffed mobile emergency care unit (MECU) manned with a physician specialist in anesthesiology and an EMT were added to the prehospital emergency medical system [6]. This unit serves as a second tier providing prehospital advanced medical treatment exceeding the competences of the EMTs (High-velocity car crash, absence of breathing, drowning etc.) (see Table 2) [6].

**Table 1:** In-hospital characteristics of shock (2000–2011)

			Overall mortality, n (%)	
Specialty/Department	n (%)	Duration of admission in days (mean)	7-days	90-days
Emergency Department	605 (36.8)	0.3	125 (20.7)	203 (33.6)
General Internal Medicine	169 (10.3)	8.6	52 (30.8)	81 (47.9)
Cardiology	160 (9.7)	5.7	44 (27.5)	71 (44.4)
Gastroenterology	134 (8.1)	9.1	27 (20.2)	45 (33.6)
Geriatriology	118 (7.2)	10.1	24 (20.3)	64 (54.2)
General Surgery	98 (6.0)	9.0	23 (23.5)	46 (46.9)
Orthopedic Surgery	55 (3.3)	13.7	9 (16.4)	20 (36.4)
Pulmonology	53 (3.2)	10.2	14 (26.4)	27 (50.9)
Endocrinology	50 (3.0)	6.8	10 (20.0)	23 (46.0)
Infectious Diseases	47 (2.9)	9.5	12 (25.5)	21 (44.7)
Heart, Pulmonary and vascular Surgery	44 (2.7)	8.9	17 (38.6)	22 (50.0)
Neurology	41 (2.5)	18.5	9 (22.0)	13 (31.7)
Nephrology	17 (1.0)	4.4	7 (41.2)	10 (58.8)
Hematology	10 (0.6)	13.3	2 (20.0)	4 (40.0)
Rheumatology	10 (0.6)	13.8	0 (0.0)	4 (40.0)
Oncology	8 (0.5)	6.8	2 (25.0)	4 (50.0)
Urology	8 (0.5)	9.1	1 (12.5)	4 (50.0)
Otorhinolaryngology	6 (0.4)	5.7	0 (0.0)	0 (0.0)
Neurosurgery	5 (0.3)	12.2	2 (40.0)	3 (60.0)
Plastic Surgery	5 (0.3)	17.2	0 (0.0)	2 (40.0)
Hospice	3 (0.2)	28.0	0 (0.0)	3 (100.0)
Total	1.646 (100.0)	5.9	380 (23.1)	670 (40.7)

**Table 2:** Prehospital and in-hospital characteristics of shock (2007–2011)

		MECU, n (%)*						Overall mortality, n (%)	
Specialty/Department	ED contacts, n (%)	Prehospital contacts, n (%)	Intravenous fluid therapy	Intravenous vasopressor therapy	Mechanical ventilation	Cardiac arrest	ICU admission, n (%)	7-days	90-days
Emergency Department	209 (27.0)	39 (18.7)	16 (7.7)	17 (2.8)	10 (4.8)	4 (1.9)	9 (4.3)	47 (22.5)	70 (33.5)
General Internal Medicine	36 (5.0)	2 (5.6)	1 (2.8)	1 (0.6)	1 (2.8)	1 (2.8)	14 (38.9)	10 (27.8)	14 (38.9)
Cardiology	79 (10.0)	19 (24.1)	11 (13.9)	11 (6.9)	12 (15.2)	10 (12.7)	22 (27.9)	26 (32.9)	40 (50.6)
Gastroenterology	74 (9.0)	11 (14.9)	6 (8.1)	7 (5.2)	4 (5.4)	0 (0.0)	18 (24.3)	14 (18.9)	27 (36.5)
Geriatriology	81 (10.0)	17 (21.0)	5 (6.2)	4 (3.4)	5 (6.2)	2 (2.5)	3 (3.7)	16 (19.8)	43 (53.1)
General Surgery	53 (7.0)	5 (9.4)	3 (5.7)	5 (5.1)	1 (1.9)	1 (1.9)	18 (34.0)	14 (26.4)	26 (49.1)
Orthopedic Surgery	35 (4.0)	4 (11.4)	3 (8.6)	3 (5.5)	0 (0.0)	0 (0.0)	8 (22.9)	6 (17.1)	13 (37.1)
Pulmonology	53 (7.0)	12 (22.6)	11 (20.8)	11 (20.8)	6 (11.3)	2 (3.8)	16 (30.2)	14 (26.4)	27 (50.9)
Endocrinology	29 (4.0)	2 (6.9)	1 (3.5)	1 (2.0)	0 (0.0)	0 (0.0)	3 (10.3)	2 (6.9)	9 (31.0)
Infectious Diseases	47 (6.0)	17 (36.2)	8 (17.0)	9 (19.2)	5 (10.6)	0 (0.0)	25 (53.2)	12 (25.5)	21 (44.7)
Heart, Pulmonary and vascular Surgery	23 (3.0)	5 (21.7)	4 (17.4)	5 (11.4)	2 (8.7)	0 (0.0)	12 (52.2)	8 (34.8)	9 (39.1)
Neurology	17 (2.0)	2 (15.4)	4 (23.53)	4 (9.8)	2 (11.8)	0 (0.0)	7 (41.2)	1 (5.9)	2 (11.8)
Nephrology	13 (2.0)	8 (47.1)	1 (7.7)	1 (9.8)	0 (0.0)	0 (0.0)	4 (30.8)	6 (46.2)	8 (61.5)
Hematology	4 (1.0)	0 (0.0)	0 (0.0)	0 (0.0)	0 (0.0)	0 (0.0)	1 (25.0)	0 (0.0)	0 (0.0)
Rheumatology	10 (1.0)	1 (10.0)	1 (10.0)	1 (10.0)	1 (10.0)	0 (0.0)	2 (20.0)	0 (0.0)	4 (40.0)
Oncology	6 (1.0)	2 (33.3)	1 (16.7)	1 (12.5)	0 (0.0)	0 (0.0)	1 (16.7)	2 (33.3)	4 (66.8)
Urology	2 (0.0)	0 (0.0)	0 (0.0)	0 (0.0)	0 (0.0)	0 (0.0)	0 (0.0)	1 (12.5)	1 (50.0)
Otorhinolaryngology	5 (1.0)	0 (0.0)	0 (0.0)	1 (16.7)	0 (0.0)	0 (0.0)	1 (20.0)	0 (0.0)	0 (0.0)
Neurosurgery	5 (1.0)	4 (80.0)	4 (80.0)	4 (80.0)	3 (60.0)	1 (20.0)	5 (100.0)	2 (40.0)	3 (60.0)
Plastic Surgery	2 (0.0)	0 (0.0)	0 (0.0)	0 (0.0)	0 (0.0)	0 (0.0)	0 (0.0)	0 (0.0)	1 (50.0)
Hospice	1 (0.0)	0 (0.0)	0 (0.0)	0 (0.0)	0 (0.0)	0 (0.0)	0 (0.0)	0 (0.0)	1 (100.0)
Total	784 (100.0)	150 (19.1)	80 (10.2)	42 (5.4)	52 (6.6)	21 (2.7)	169 (21.6)	180 (23.0)	323 (41.2)

*ED* emergency department, *ICU* intensive care unit, *MECU* Physician-staffed mobile emergency care units

*Data on MECU transportation and ICU admission available from 2007 to 2011

In 2009 the Adaptive process triage (ADAPT) was implemented in the ED at Odense University Hospital and is the most commonly used triage system in Denmark [7]. Prior to 2009, the severity and urgency of a patient’s condition were evaluated by an experienced nurse who measured vital values in accordance with a clinical judgment followed by blood test analysis based at a doctors prescription. Patients suffering minor complaints (e.g. sprained ankle, insect bite without systemic reactions etc.) had usually not their vital values measured or blood test analysis performed.

### Participants

Eligible patients were all patients aged ≥18 years presenting to the ED with a systolic blood pressure (SBP) ≤100 mmHg registered within 3 h upon arrival during the study period. We chose to use a higher threshold (100 mmHg) of hypotension than the traditional 90 mmHg. This decision was based on increasing evidence advocating for a redefinition of arterial hypotension [8–11], which we also have underlined in a recent study within our research unit [12]. The primary date of contact defined the index date. If a patient had multiple ED visits with hypotension over the study period, only the first was included in the cohort. Patients residing outside the hospitals catchment area at the time of contact and patients without a Danish personal identification number were excluded. Patients who had visited the ED between 1 of January 1998 and 1 of January 2000 with hypotension were excluded to minimize left sided censoring. The background population, from which patients were retrieved, was all adult (≥18 years) Danish citizens living in the hospitals catchment area. Patients were followed from index date until the date of death, emigration, December 31, 2011, or completion of 90 days follow-up, whichever came first.

### Data sources and processing

#### Database

Since 1996 all patients records from the ED are registered electronically and available as patients record notes from the primary contact. The record notes are available in structured text-format, in which vital parameters are consistently stated, including measured SBP and heart rate (HR) and time of admission. By electronic screening it was possible to identify and retrieve information on all patients with the unique registered value of SBP and HR. The principle of free-text search has been validated in the context of extracting numerical data, including blood pressure recordings [13]. The data extraction process used has previously been validated in 500 random ED notes to have a sensitivity of 95.8 % (95 % CI [91.2, 98.5]) and a specificity of 100 % (95 % CI [99.0, 100]) for retrieving correct SBP [12, 14].

### Population-based registers

In Denmark every Danish citizen has free individual access to tax-supported health care provided by The Danish National Health Service. At birth the Danish Civil Registration system (CRS) assigns a unique 10-digit civil personal registry number (PRN-number) to each Danish citizen and to residents upon immigration since 1968. This unique PRN-number enables accurate linkage of the Danish national registers [15]. True population-based studies are hereby possible as all patient contacts are registered and linked between all Danish registries using the patients unique PRN-number.

#### The Danish National Patient Registry

Since 1995 the Danish National Patient Registry has been covering all in-patient and out-patient clinic contacts at hospitals in Denmark assembling data regarding dates of admission and discharge, admitting departments, and all primary and secondary discharge diagnoses (ICD-10 code system) from hospitals [16]. Since 1994 every patient admission, discharge and procedures performed has been registered according to the ICD-10 code system [17]. At discharge every patient is assigned one primary diagnosis and up to 20 secondary diagnoses. Data on municipality of residence, migration-, vital status, and date of birth were retrieved from The Danish Civil Registration System [15].

### Outcome measures, exposure and possible confounders

We defined shock as the presence SBP ≤100 mmHg [12] and ≥1 organ failures.

The following organ failures were included: Cardiovascular, Renal, Coagulation and Hepatic. Biochemical variables (creatine, bilirubin, platelets and INR (international normalized ratio)) registered 180 days before and 1 day after the index date was used to identify renal, hepatic and coagulative failure (see Appendix for details). We used the Shock Index (SI) as a measure of cardiovascular failure. SI is calculated as the ratio of heart rate to SBP and included as a categorical variable (<0.7, 0.7–1, ≥1) [18]. We defined cardiovascular failure as SI ≥1. SBP was measured with an automated oscillometric device or manual cuff and sphygmomanometer. Heart rate was measured with ECG, palpation or pulse oximetry. The primary outcome was all-cause mortality within 7-days of the index date. Secondary outcomes were 90-day mortality as well as factors associated with death and annual IRs during the study period. The primary exposure variables were the first recorded SBP ≤100 mmHg at presentation, registered within 3 h upon arrival and the presence of ≥1 organ failures. As the laboratorial analysis of biochemical variables could exceed 3 h (due to busy hours, crowding etc.) we computed organ failures based on variables registered within 24 h after arrival to the ED.

We also included information on the additional covariates; gender, age, SBP level (90> SBP ≤100 mmHg, 80> SBP ≤90 mmHg, SBP ≤80 mmHg) and Charlson comorbidity index. The latter was used as a proxy for comorbid illness [19]. We used discharge diagnoses from the previous 10 years in order to generate the Charlson comorbidity index (CCI; 0, 1–2, >2) for each enrolled patient upon the index contact date [19].

### Statistical analysis

We presented continuous and categorical data as medians (interquartile range (IQR)) and numbers (%), respectively.

#### Incidence rates

The crude annual IRs were calculated as the number of IRs per 100,000 person-years at risk (pyar) (age ≥18 years) with the corresponding 95 % confidence intervals (95 % CI) assuming a Poisson distribution. The annual IRs were adjusted using direct standardization to the sex- and age distribution of the municipalities of the EDs catchment area midyear population in the year 2000. The population was defined as contributing to one pyar per resident per year in the analyses (http://www.statistikbanken.dk/FOLK1, http://www.statistikbanken.dk/BEF6, http://www.statistikbanken.dk/BEF607). The incidence rates were estimated and analyzed using a Poisson regression model. Sex, age group, calendar time in years, and interaction between age group and sex were used in the adjusted model. Calender time was entered in the model as a continuous variable. Age was divided into four predefined age intervals: 18–39, 40–64, 65–84 and ≥85 years. The Poisson model was assessed using the Hosmere Lemeshow goodness-of-fit test.

#### All-cause mortality analysis

All-cause mortality was presented in a Kaplan-Meier plot and comparison between survival curves was tested using log-rank test. All-cause mortality proportions were reported at 7-, and 90-days after the index date. Risk factors for all-cause mortality were evaluated by Cox regression and presented as unadjusted and adjusted hazard ratios (HRs) with 95 % confidence intervals (CIs) for time periods 0 to 7-days and 8 to 90-days. The models were adjusted for the following predefined variables: sex, age, Charlson comorbidity index, and number of organ failures (1, 2 and ≥3).

Interaction between covariates where examined on all covariates and none were included. We included age as a continuous variable after testing the assumptions of linearity using a restricted cubic spline with 5 knots. Furthermore, the proportional hazards assumption was checked by visual inspection of log**–**log plots of survival using the scaled Schoenfeld residuals. We finally tested the model using Cox-Snell residuals and found the model fitting the data well. Cuzick’s test was used for trends in annual mortality.

Statistical analyses were performed using Stata version 13.1 (Stata Corporation LP ®, Texas, USA).

## Results

### Participants

**Fig. 1:**
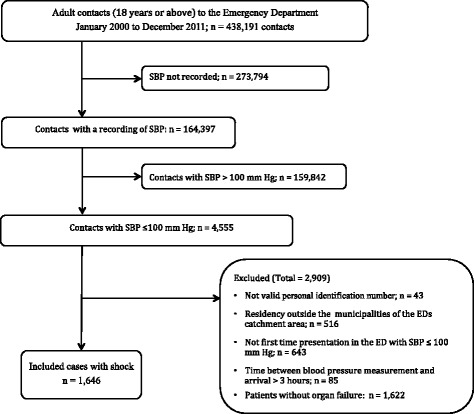
Flow chart of patients recruited to the study

Of 438,191 ED contacts a total 1646 (0.4 %) patients presented with shock and were included in the analysis. Reasons for exclusions are presented in Fig. 1 and baseline characteristics in Table 3.

**Table 3:** Baseline characteristics at time of arrival to the ED^a^

Variable	Total (%)
N (%)	1,646 (100 %)
Age in years, Median (IQR)	71 (56–80)
Sex (%)	
Male	885 (53.8 %)
Female	761 (46.2 %)
Age in age groups, yr (%)	
18–39	146 (8.9 %)
40–64	496 (30.1 %)
65–84	745 (45.3 %)
85+	259 (15.7 %)
Charlson Comorbidity Index (%)	
0	495 (30.1 %)
1	627 (38.1 %)
>2	524 (31.8 %)
Vital variables	
Systolic blood pressure, Median (IQR)	88 (80–94)
Diastolic blood pressure, Median (IQR)	53 (44–62)
Heart rate, Median (IQR)	100 (85–114)
Shock Index (SI), n (%)	
SI, Median (IQR)	1.2 (1.0–1.3)
SI ≤0.7	94 (5.9)
0.7> SI ≤1	267 (16.6 %)
SI >1.0	1,245 (77.5 %)
Number of organ failures, n (%)	
1	1,155 (70.2 %)
2	389 (23.6 %)
3+	114 (6.2 %)
Site of organ failure (%)	
Cardiovascular	1,245 (76.6 %)
Renal	382 (23.2 %)
Coagulation	545 (33.1 %)
Hepatic	78 (4.7 %)

^a^Values expressed as total number (fraction) and medians [25 percentile-75 percentile] as appropriate

The median SBP on presentation was 88 mmHg (IQR, 80–94 mmHg) with a median SI of 1.2 (IQR, 1.0–1.3). The most frequent organ failure was cardiovascular present in 76.6 % (1245) of the patients). One organ failure was present in 70.2 % (1155), 23.6 % (389) had 2 and 6.2 % (114) had ≥3 failures (Table 3). The proportion of admittance to non-surgical specialties was 49.8 % (820), whereas 36.8 % (605) patients were evaluated exclusively in the ED (Table 1). In the period 2007–2011, 784 patients were assessed in the ED of which 19.7 % (150) had a prehospital contact to a physician (MECU), and 21.6 % (169) were admitted to the ICU (Table 2).

### Incidence of shock

**Fig. 2:**
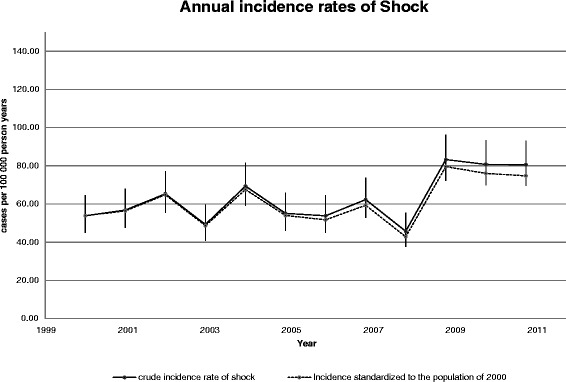
Annual incidence rate during 2000–2011. The crude annual incidence rates of shock from 2000 to 2011 and the standardized incidence rate to the population of the EDs cathment area in 2000 (using direct standardization on sex and ten-year age bands). Bars indicate the 95 % confidence interval based on a Poisson distribution

**Fig. 3:**
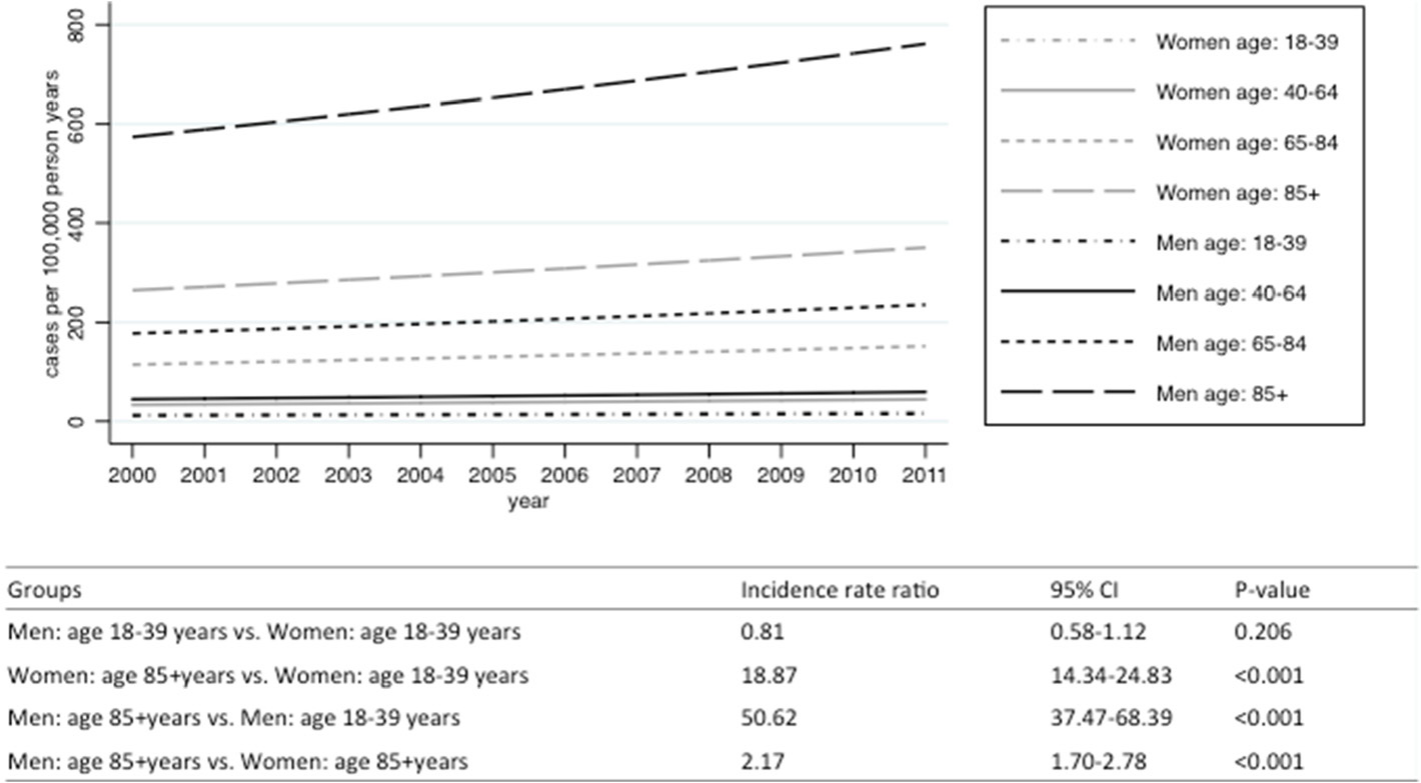
Estimated incidence rates stratified by sex and age group from 2000 to 2011. Incidence rates estimated on the basis of a Poisson model adjusting for sex, age group, interaction between sex and age group, and calendar years. The table is showing the corresponding estimated incidence rate ratios with 95 % confidence intervals (95 % CI)

The yearly crude IR are shown in Fig. 2 together with the standardized IR. The mean annual IR of shock was 63.2 cases per 100,000 pyar (95 % CI: 60.2–66.3). The IR increased from 53.8 to 80.6 cases per 100,000 pyar, during the period 2000–2011, with an average adjusted annual increase of 2.6 % (95 % CI: 1.1–4.2). The average annual increase using standardized estimates was 2.7 % (95 % CI: 1.0–4.9). The estimated incidence rates stratified by sex and age group with incidence rate ratios are shown in Fig. 3. Men aged 85+ had a fifty-time higher IR than men aged 18–39 years.

### Mortality among patients with shock

**Fig. 4:**
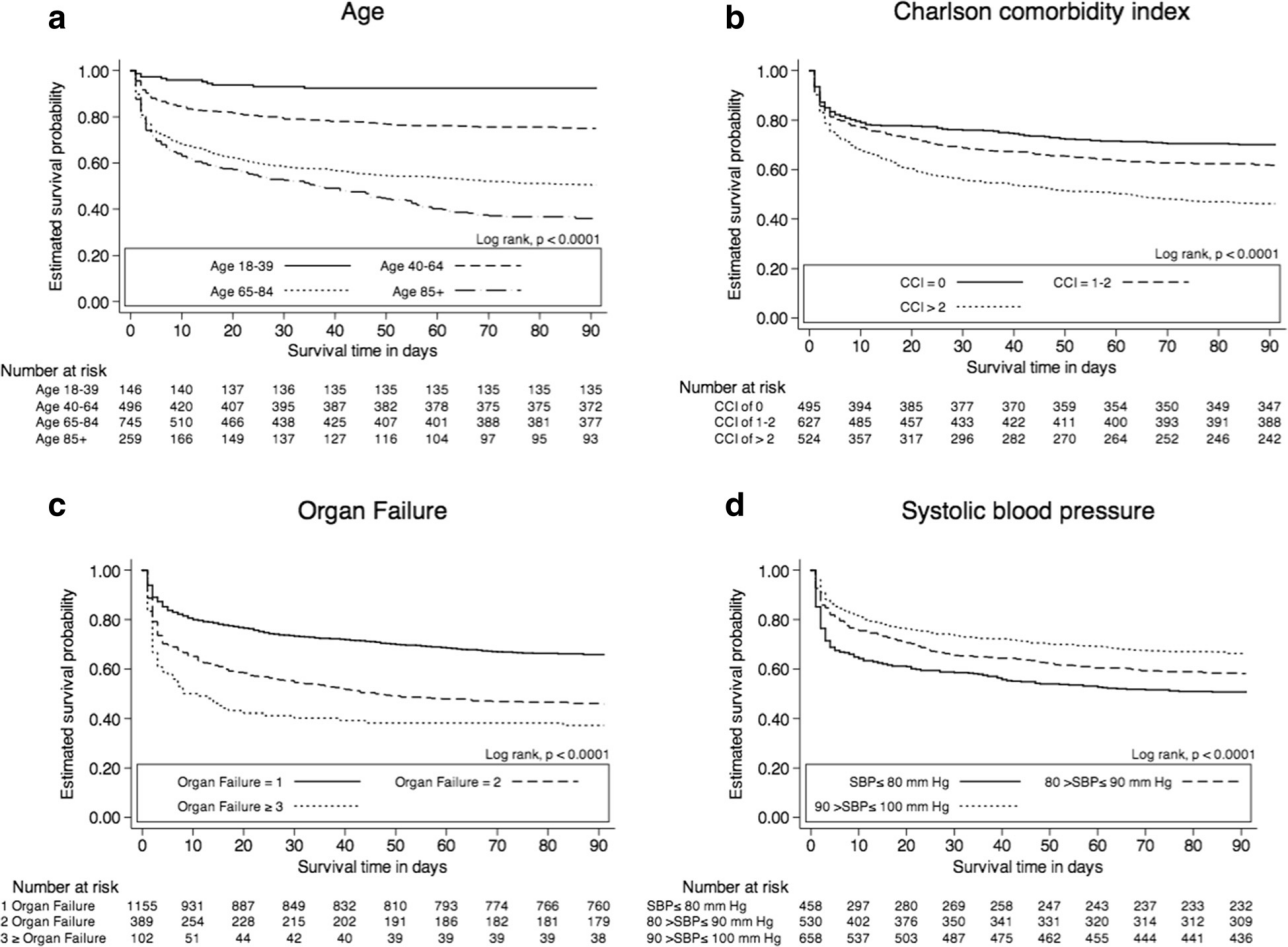
Kaplan-Meier curves illustrating overall 90-day survival according to age (**a**), Charlson comorbidity index (**b**), organ failures (**c**) and systolic blood pressure levels (**d**). Below the curves are listed the number at risk at corresponding intervals in survival time

Among patients presenting with shock 380/1646 died within 7 days ((23.1 % (95 % CI: 21.1–25.1)) and a total 670/1646 died within 90 days (40.7 % (95 % CI: 38.3–43.1),) (Table 4). Trend analysis of the annual 7-, and 90-day mortality proportions did not show any significant change during the entire observation period (7-day mortality: *P*_*trend*_ = 0.513 and 90-day mortality: *P*_*trend*_ = 0.674). Kaplan-Meier curves are shown in Fig. 4 with the overall estimated probability of 90-day survival stratified into age (Fig. 4a), Charlson comorbidity index (Fig. 4b), organ failures (Fig. 4c) and systolic blood pressure (Fig. 4d).

**Table 4:** Prognostic factors of death in patients presenting with shock at presentation to the ED – Cox regression

		0–7 days					8–90 days				
	N, total (%)	N, died (%)	Crude HR (95 % CI)	*p* Value	Adjusted HR (95 % CI) ^a^	*p* Value	N, died (%)	Crude HR (95 % CI)	*p* Value	Adjusted HR (95 % CI) ^a^	*p* Value
Gender											
Female (reference)	761 (46.2)	167 (21.9)	1		1		133 (22.4)	1		1	
Male	885 (53.8)	213 (24.1)	1.12 (0.91–1.37)	0.289	1.10 (0.90–1.35)	0.354	157 (23.4)	1.05 (0.83–1.32)	0.686	1.03 (0.82–1.30)	0.813
Age (continous)			1.03 (1.03–1.04)	<0.001	1.03 (1.03–1.04)	<0.001		1.04 (1.04–1.05)	<0.001	1.04 (1.03–1.05)	<0.001
Comorbidity level											
0 (reference)	495 (30.1)	95 (19.2)	1		1		53 (13.3)	1		1	
1 to 2	627 (38.1)	134 (21.4)	1.13 (0.87–1.47)	0.372	0.85 (0.65–1.11)	0.229	106 (21.5)	1.71 (1.23–2.37)	0.001	1.23 (0.88–1.71)	0.229
>2	524 (31.8)	151 (28.8)	1.56 (1.21–2.02)	0.001	1.10 (0.85–1.43)	0.470	131 (22.9)	3.06 (2.22–4.20)	<0.001	2.03 (1.47–2.80)	<0.001
Number of organ failures											
1 (reference)	1,155 (70.2)	206 (17.8)	1		1		189 (19.9)	1		1	
2	389 (23.6)	126 (32.4)	1.96 (1.57–2.44)	<0.001	1.90 (1.53–2.38)	<0.001	85 (32.3)	1.78 (1.37–2.30)	<0.001	1.76 (1.36–2.28)	<0.001
3+	102 (6.2)	48 (47.1)	3.08 (2.25–4.22)	<0.001	3.13 (2.28–4.30)	<0.001	16 (29.6)	1.67 (1.00–2.79)	0.048	1.75 (1.05–2.92)	0.033

^a^Cox proportional hazard model adjusted for sex, age as a continuous variable, Charlson comorbidity level (0, 1–2, >2) and number of organ failures. Patients who died during the first 7 days after admission were excluded from the analyses of 8- to 90-day mortality

### Prognostic factors of death among patients with shock

In the multivariate analysis patients with organ failures of 2 (HR = 1.90 (95 % CI, 1.53–2.38)) and ≥3 (HR = 3.13 (95 % CI, 2.28–4.30)) had a higher rate as compared to the reference within 0–7 days. Concordantly, patients with 2 failures (HR = 1.76 (95 % CI, 1.36–2.28)) and ≥3 (HR = 1.75 (95 % CI, 1.05–2.92)) failures had a higher rate as compared to the reference within 8–90 days. Age depicted an increased risk of death within 7 days, whereas comorbidity was not a significant predictor. Within 8–90 days, predictors; age, and Charlson comorbidity index >2 were associated with increased risk of death (Table 4).

## Discussion

In the present study, we have described a well-defined cohort of patients suffering shock upon arrival to the ED. The results reveal that shock is frequently encountered in the ED and is associated with a substantial mortality.

We found the prevalence of hypotensive shock to be 0.4 % (1646/438,191), corresponding to a mean annual incidence of 63.2/100,000 pyar (95 % CI: 60.2–66.3). The overall IR of registered shock increased during 2009–2011 compared to the previous years. This increase could be attributed to the introduction of the ADAPT algorithm in our ED in 2009 by which the identification of critically ill patients became more standardized, as compared to the years before. We found shock to be most common among the elderly with a higher incidence among men. The gender specific difference in the IR could be due to the fact that men in general have more comorbidity than women. Whether increased awareness across etiologies during this period (surviving sepsis campaign and percutaneous coronary intervention of myocardial infarction) is of importance remains to be explored. However, the present finding suggests shock to be as frequent as an ED presentation of ST-elevation myocardial infarction [20]. As opposed to myocardial infarction, research investigating characteristics of ED shock have been limited [21].

This cohort further demonstrates shock as a critical finding carrying a 7-, and 90-day mortality of 23.1 and 40.7 %, respectively. Although it is well accepted, that shock associates poor prognosis, the mortality reported here exceeds previous reported estimates of shock in the ICU and ED setting [4, 5, 22, 23]. Comparing mortality outcomes depends largely on setting of research and the underlying etiology. Prior studies typically evaluate outcomes in patients with a single etiology of shock, whereby extrapolation to an open general ED is somewhat arbitrary. Although prognosis have improved across etiologies of shock, mortality continuous to be critically high [1]. Studies investigating non-traumatic shock report inhospital mortality of 16–25 % [4, 5, 22] in the ED, whereas mortality estimates in the ICU setting is 38 % [23]. For patients with septic- or cardiogenic shock mortality is 32 % [24] and 34 % [25], respectively. Traumatic shock carries a somewhat lower mortality of 16 % [26]. The estimates from our study should be interpreted in the context of the undifferentiated population from which they are derived, as opposed to the selected patient populations in the ICU’s or specialized units with well-defined etiologies.

In the current study, severity of shock (based on the number of organ failures) and age appears to be the most important determinants of clinical outcome within the first week after presentation. Conditional upon surviving the first week, the underlying comorbid burden is an important factor for death within 8–90 days as well as the number of organ failures and age. These findings are in line with previous studies investigating critical illness and outcomes, suggesting multiple organ dysfunction and multiple comorbidities to depict poor outcomes [27].

Despite technical improvement in diagnostics and advances in treatment, during the past decades, shock is still a critical finding in the acute medical care and ED setting. Steps to improve outcome have been implemented in which acute medical personal identify life-threatening conditions, mobilize critical resources, and initiate relevant therapy. Within specific groups of critically ill populations, goal directed team approaches have been successful (trauma, cardiac arrest, and sepsis). Patient suffering undifferentiated shock may benefit from a similar approach [28]. However, reducing time to recognition is a critical aspect of caring for patients suffering shock. Clinical recognition of shock is traditionally based on vital sign abnormalities. Measurement of SBP and heart rate is a commonly used clinical practice to assess the circulatory state of acutely ill patients. The presence of hypotension often signifies overt shock and even a transient presentation of hypotension should alert the clinician to warrant careful attention and evaluation for the presence of shock. Future studies should refine the diagnostic process of recognizing shock in the ED. Moreover, exploring baseline etiological characteristics of undifferentiated shock at presentation in the ED are needed.

### Study strengths and limitations

In this study, we analyzed a large cohort of acutely ill, undifferentiated patients arriving to the ED. We had no loss to follow-up do to the unique personal registration numbers in Denmark. The Danish public healthcare system, with a complete, independently and prospectively recorded medical history, made it possible to identify all included patients in the population-based registries. We were hereby able to compute robust estimates on incidence, all-course mortality and predictive factors for death.

The blood pressure measurements were registered prospectively and as a routine documentation and triage in the ED population. In order to avoid possible overestimation of the IR, we excluded patient with residency outside the catchment area and a previously reported admission with SBP ≤100 mmHg in the years 1998–99. To minimize bias from repeated measurements we used the first contact with shock, within the study period.

There are limitations and possible bias that must be kept in mind when interpreting our findings. This was a single-center, retrospective study from a University Hospital ED serving a well-defined catchment area and is the primary and only hospital in this area of Denmark. The results may, however, not necessarily be generalized to other hospitals. Although our ED is the only on serving this part of Denmark, we are not able to adjust for patients living in our catchment area, who have had contact to other hospitals. However, in order minimize this proportion (*n* = 516, Fig. 1) we excluded patients living in municipalities outside of our ED catchment.

An important limitation is the proportion of patients who were not included as a SBP was not measured upon arrival (*n* = 273,774). These patients suffered minor complaints and the triaging nurses did not measure SBP based on a clinical judgment. These circumstances also apply for the proportion of patients, who did not have blood test performed upon arrival (*n* = 698). However, the retrospective data at hand are a reflection of the everyday procedures in our ED and not necessarily collected for research purposes.

We defined hypotension as SBP ≤100 mmHg, based on increasing evidence supporting a higher threshold, as opposed to the traditional 90 mmHg [11, 12]. We used the first recorded SBP value registered and did not have the possibility to examine individual dynamic trends by serial measurements. Although a more detailed definition of hypotension taking into account a patient’s baseline blood pressure as well as repeated measurements in the ED would be ideal, it was not feasible in this study. However, a single measurement approach is a common clinical applied triage method in emergency medicine settings.

Another important limitation is the number of organ failures defining our cohort. Metabolic failure was not included, as arterial punctures were not systematically collected. Moreover, respiratory frequencies and Glasgow Coma Scale were not consistently registered, whereby organ failures related to the respiratory system, and failure of the central nervous system were not included. We used a Shock Index ≥1 to define cardiovascular failure, as this index has been shown to prognosticate outcome across several etiologies of shock and critical illnesses [18, 29–36]. Ideally, cardiac output measurements would have been desirable but not feasible based on the present design. As not all variables for assessing organ failure were available, the incidence rate and the mortality outcomes should be interpreted bearing this in mind.

Furthermore, we acknowledge the presence of a physician in the prehospital setting (MECU) (from 2006 and onwards) could induce referral bias, as certain “high-risk” patients are prone to be transported directly from the pre-hospital setting to the operational theater or ICU, and thereby by-pass the ED. Moreover, in the period 2000–2008 (prior to the implementation of the ADAPT algorithm) blood pressure and blood test were taken only if the acute care ED personal deemed it appropriate whereby our outcomes could be susceptible to selection bias.

Lastly, a significant proportion of patients were evaluated in the ED and either discharged, died or admitted to the ICU (Table 2). The later could be susceptible to information bias, as the registration of ICU admission directly from the ED was not consistently documented during the period of observation. Although limited, we had missing values on covariates; ICD-codes (2 patients) and HR (40 patients).

## Conclusion

Shock is present in 0.4 % of ED encounters, with a mean annual IR of 63.2/100,000 pyar (95 % CI: 60.2–66.3) carrying a substantial 7-day, and 90-day all-cause mortality. Age and increasing number of organ failures are important prognostic factors associated with increased risk 7 days after ED presentation, whereas age, comorbidity and number of organ failures are prognostic factors at 8 to 90 days.

## Appendix

### Definition of Organ failure

**Organ failure was defined as the occurrence of one of the following affections based on variables registered within 180 days before and 1 day after the index date:**

**Renal:**

S-creatinine >177 μmol/L and >100 μmol/L S-creatinine increase from earlier S-creatinine

OR

S-creatinine >177 μmol/L and earlier S-creatinine <130 μmol/L or never previously registered

**Hepatic:**

S-bilirubin >42 μmol/L and earlier S-bilirubin <43 μmol/L or never previously registered

**Coagulation:**

Platelet count <101*10^9^/L and earlier platelet count >100*10^9^/L or never previously registered

OR

INR >1.59 and earlier INR <1.60 or never previously registered

## References

[1] Vincent JL, De Backer D (2013). Circulatory shock. N Engl J Med.

[2] Torio CM, Andrews RM. National Inpatient Hospital Costs: The Most Expensive Conditions by Payer, 2011. Rockville (MD): Healthcare Cost and Utilization Project (HCUP) Statistical Brief #160; 2006. Available at http://www.hcupus.ahrq.gov/reports/statbriefs/sb160.jsp. 24199255

[3] Havel C, Arrich J, Losert H, Gamper G, Mullner M, Herkner H (2011). Vasopressors for hypotensive shock. Cochrane Database Syst Rev.

[4] Jones AE, Tayal VS, Sullivan DM, Kline JA (2004). Randomized, controlled trial of immediate versus delayed goal-directed ultrasound to identify the cause of nontraumatic hypotension in emergency department patients. Crit Care Med.

[5] Moore CL, Rose GA, Tayal VS, Sullivan DM, Arrowood JA, Kline JA (2002). Determination of left ventricular function by emergency physician echocardiography of hypotensive patients. Acad Emerg Med Off J Soc Acad Emerg Med.

[6] Mikkelsen S, Kruger AJ, Zwisler ST, Brochner AC (2015). Outcome following physician supervised prehospital resuscitation: a retrospective study. BMJ Open.

[7] Lindberg SO, Lerche la Cour J, Folkestad L, Hallas P, Brabrand M (2011). The use of triage in Danish emergency departments. Dan Med Bull.

[8] Seymour CW, Cooke CR, Heckbert SR, Copass MK, Yealy DM, Spertus JA (2013). Prehospital systolic blood pressure thresholds: a community-based outcomes study. Acad Emerg Med Off J Soc Acad Emerg Med.

[9] Eastridge BJ, Salinas J, McManus JG, Blackburn L, Bugler EM, Cooke WH (2007). Hypotension begins at 110 mm Hg: redefining “hypotension” with data. J Trauma.

[10] Bruns B, Gentilello L, Elliott A, Shafi S (2008). Prehospital hypotension redefined. J Trauma.

[11] Jones AE, Yiannibas V, Johnson C, Kline JA (2006). Emergency department hypotension predicts sudden unexpected in-hospital mortality: a prospective cohort study. Chest.

[12] Kristensen AK, Holler JG, Mikkelsen S, Hallas J, Lassen A (2015). Systolic blood pressure and short-term mortality in the emergency department and prehospital setting: a hospital-based cohort study. Crit Care.

[13] Turchin A, Kolatkar NS, Grant RW, Makhni EC, Pendergrass ML, Einbinder JS (2006). Using regular expressions to abstract blood pressure and treatment intensification information from the text of physician notes. J Am Med Inform Assoc.

[14] Kristensen AK, Holler JG, Hallas J, Lassen A, Shapiro NI. Is Shock Index a Valid Predictor of Mortality in Emergency Department Patients With Hypertension, Diabetes, High Age, or Receipt of beta- or Calcium Channel Blockers? Annals of emergency medicine 2015. doi: 10.1016/j.annemergmed.2015.05.020 10.1016/j.annemergmed.2015.05.020 26144893

[15] Schmidt M, Pedersen L, Sorensen HT (2014). The Danish Civil Registration System as a tool in epidemiology. Eur J Epidemiol.

[16] Lynge E, Sandegaard JL, Rebolj M (2011). The Danish National Patient Register. Scand J Public Health.

[17] Andersen TF, Madsen M, Jorgensen J, Mellemkjoer L, Olsen JH (1999). The Danish National Hospital Register. A valuable source of data for modern health sciences. Dan Med Bull.

[18] Rady MY, Smithline HA, Blake H, Nowak R, Rivers E (1994). A comparison of the shock index and conventional vital signs to identify acute, critical illness in the emergency department. Ann Emerg Med.

[19] Charlson ME, Pompei P, Ales KL, MacKenzie CR (1987). A new method of classifying prognostic comorbidity in longitudinal studies: development and validation. J Chronic Dis.

[20] Ward MJ, Kripalani S, Zhu Y, Storrow AB, Dittus RS, Harrell FE (2015). Incidence of emergency department visits for ST-elevation myocardial infarction in a recent six-year period in the United States. Am J Cardiol.

[21] Holler JG, Bech CN, Henriksen DP, Mikkelsen S, Pedersen C, Lassen AT (2015). Nontraumatic Hypotension and Shock in the Emergency Department and the Prehospital setting, Prevalence, Etiology, and Mortality: A Systematic Review. PLoS One.

[22] Jones AE, Stiell IG, Nesbitt LP, Spaite DW, Hasan N, Watts BA (2004). Nontraumatic out-of-hospital hypotension predicts inhospital mortality. Ann Emerg Med.

[23] Sakr Y, Reinhart K, Vincent JL, Sprung CL, Moreno R, Ranieri VM (2006). Does dopamine administration in shock influence outcome? Results of the Sepsis Occurrence in Acutely Ill Patients (SOAP) Study. Crit Care Med.

[24] Levy MM, Artigas A, Phillips GS, Rhodes A, Beale R, Osborn T (2012). Outcomes of the Surviving Sepsis Campaign in intensive care units in the USA and Europe: a prospective cohort study. Lancet Infect Dis.

[25] Kolte D, Khera S, Aronow WS, Mujib M, Palaniswamy C, Sule S (2014). Trends in incidence, management, and outcomes of cardiogenic shock complicating ST-elevation myocardial infarction in the United States. J Am Heart Assoc.

[26] Cannon CM, Braxton CC, Kling-Smith M, Mahnken JD, Carlton E, Moncure M (2009). Utility of the shock index in predicting mortality in traumatically injured patients. J Trauma.

[27] Esper AM, Martin GS (2011). The impact of comorbid [corrected] conditions on critical illness. Crit Care Med.

[28] Sebat F, Musthafa AA, Johnson D, Kramer AA, Shoffner D, Eliason M (2007). Effect of a rapid response system for patients in shock on time to treatment and mortality during 5 years. Crit Care Med.

[29] Rady MY, Rivers EP, Martin GB, Smithline H, Appelton T, Nowak RM (1992). Continuous central venous oximetry and shock index in the emergency department: use in the evaluation of clinical shock. Am J Emerg Med.

[30] Yussof SJ, Zakaria MI, Mohamed FL, Bujang MA, Lakshmanan S, Asaari AH (2012). Value of Shock Index in prognosticating the short-term outcome of death for patients presenting with severe sepsis and septic shock in the emergency department. Med J Malaysia.

[31] Mutschler M, Nienaber U, Munzberg M, Wolfl C, Schoechl H, Paffrath T (2013). The Shock Index revisited - a fast guide to transfusion requirement? A retrospective analysis on 21,853 patients derived from the TraumaRegister DGU. Crit Care.

[32] McNab A, Burns B, Bhullar I, Chesire D, Kerwin A (2013). An analysis of shock index as a correlate for outcomes in trauma by age group. Surgery.

[33] Toosi MS, Merlino JD, Leeper KV (2008). Prognostic value of the shock index along with transthoracic echocardiography in risk stratification of patients with acute pulmonary embolism. Am J Cardiol.

[34] Wira CR, Francis MW, Bhat S, Ehrman R, Conner D, Siegel M (2014). The shock index as a predictor of vasopressor use in emergency department patients with severe sepsis. West J Emerg Med.

[35] Berger T, Green J, Horeczko T, Hagar Y, Garg N, Suarez A (2013). Shock index and early recognition of sepsis in the emergency department: pilot study. West J Emerg Med.

[36] Olaussen A, Blackburn T, Mitra B, Fitzgerald M (2014). Review article: shock index for prediction of critical bleeding post-trauma: a systematic review. Emerg Med Australas.

[37] Vandenbroucke JP, von Elm E, Altman DG, Gotzsche PC, Mulrow CD, Pocock SJ (2007). Strengthening the Reporting of Observational Studies in Epidemiology (STROBE): explanation and elaboration. PLoS Med.

